# Preliminary Study of the Psychometric Properties of a Questionnaire to Assess Spanish Canoeists’ Perceptions of the Sport System’s Capacity for Talent Development in Women’s Canoeing

**DOI:** 10.3390/ijerph19073901

**Published:** 2022-03-25

**Authors:** Juan Carlos Guevara-Pérez, Jorge Rojo-Ramos, Santiago Gómez-Paniagua, Jorge Pérez-Gómez, José Carmelo Adsuar

**Affiliations:** 1 Faculty of Economics and Business, University of Zaragoza, 50005 Zaragoza, Spain; jguevara@unizar.es or; 2 IGOID Research Group, Department of Physical Activity and Sport Sciences, University of Castilla-La Mancha, 45071 Toledo, Spain; 3 Health Economy Motricity and Education (HEME) Research Group, Faculty of Sport Science, University of Extremadura, 10003 Caceres, Spain; 4 BioẼrgon Research Group, University of Extremadura, 10003 Caceres, Spain; sgomezpa@alumnos.unex.es; 5 Promoting a Healthy Society (PHeSO) Research Group, Faculty of Sport Sciences, University of Extremadura, 10003 Caceres, Spain; jadssal@unex.es

**Keywords:** women and sport, talent production, canoeing, Olympic Games, sport system

## Abstract

Given the importance of sport at a global level, the competitiveness of sport systems is a determining factor in attracting resources from different sectors. Competitiveness is largely measured by the athletes’ level. Therefore, the production of competitive talent is an aspect that occupies the managers of different sports systems. This study analyzed the factor structure and reliability of a questionnaire for the evaluation of the perceptions of actors of a sport (canoeing) on the ability of the national system to produce talent in one of its modalities recently incorporated in the Olympic Games (OG) of Tokyo 2021. The sample consisted of 167 individuals linked to Spanish canoeing, who responded to the questionnaire “Evaluation of the current position in canoeing-sport with regard to talent” of the International Canoe Federation (ICF). Exploratory, confirmatory and reliability factor analyses were performed. The results showed a one-dimensional factor structure composed of seven items, with good and excellent goodness-of-fit values and high reliability (McDonald’s Omega = 0.82). Thus, the ICF questionnaire can be considered a quick and easy to apply tool to analyze the perceptions about the development of talent in canoeing in order to take actions for the recruitment, promotion and development of talent.

## 1. Introduction

Few sectors have an economic and social scope comparable to sports whose coverage is currently unquestionable. At the moment, different countries and international organizations are fighting to host sporting events, in search of their numerous benefits, such as an improvement in the country’s or city’s perception or image, social and cultural benefits, poverty reduction and employment generation, promotional advantages and infrastructural developments, as examples [1,2]. It is difficult to find an event capable of capturing global attention like the Olympic Games or the World Cup. For this reason, one of the differentiating attributes of the sports phenomenon is competition, since without it, sport would be reduced to a mere recreational game [3]. Therefore, the level of competition is decisive for the media coverage of the sector and the ability to attract private capital. In this regard, there is a vast literature supporting the attractiveness of the show as a function of the competitive balance [4,5,6,7], which is difficult to achieve without the incorporation of talent in the competition system.

On the other hand, in the governmental and non-profit sphere, the competitive level is also important for countries to show the vigor of their social management through sport. One example is the preparation for the Olympic Games, where the states allocate public resources to the sector through the sports federations, which must invest in them efficiently both in the promotion and development of their activity as well as in the preparation of their athletes for international competitions. There is a great deal of research in this area that has served as a framework for the study of efficiency in the rational use of resources allocated to sport through qualitative approaches [8,9,10] or quantitative models to measure performance [11]. There are also studies focused on the effectiveness in obtaining competitive results and of the factors that lead to sporting success [12]. In any case, the search for efficiency and effectiveness in sport are open issues, for which talent development is a common input [13].

Regardless of the approach adopted by the organizations, the research requires either extensive databases or the time and effort involved in gathering information through interviews. In any case, and given that the generation of talent is a key aspect for obtaining competitive results, evaluating several instruments is appropriate for taking a first look at the capacity of a sports system to develop talent. Nevertheless, the majority of those instruments focus their evaluations on intrinsic athlete variables such as physical aspects [14], cognitive issues [15], physiological and anthropometric heritage [16] or their development process [17], carrying out a very superficial analysis of this question, since many of these theories are confronted and do not share links for a multidisciplinary approach.

In this sense, the Spanish context has served as the setting for previous investigations, shedding light on several unknowns in this area of knowledge [18,19]. Additionally, in Spain, high-level sport is considered of interest to the state [20] with the sports federations (FFDD) in collaboration with the Autonomous Communities (CCAA), being responsible for the development of high-level sport and public functions of an administrative nature and acting as collaborating agents of the Public Administration [21]. Despite the fact that canoeing is not a popular activity in Spain, it is the sport that has earned the most medals in the last three Olympic cycles and one of the disciplines with the most medals [19] in Spanish Olympic competition [22]. Therefore, canoeing is one of the oldest sports and one with the most events in the Olympic program [16], giving its strategic importance for national sports systems.

Sport’s propensity to exclude or marginalize alternative gender identities has been thoroughly documented [23], with critiques demonstrating how some sports reinforce dominant masculine standards that operate to exclude or minimize other gender identities [24]. Changes in the modern sport landscape, on the other hand, may lead to a reshaping and reimagining of gender relations in sport [25]. Women are increasingly engaging in, teaching, and administering sports [26], while male-dominated sports are seeing a significant rise in women’s participation, indicating a shift in traditional gendered ideas and views. Despite this, there is still a long way to go to achieve gender equality in the world of sport in terms of salaries, sports development at the grassroots level, publicity and opportunities [27].

For all of the above, and given the positioning of canoeing in Spain and the World, we have considered it pertinent to take a look at the actions developed in the country during the qualification and participation route of the last Olympic cycle, since the incorporation of women in canoeing was an extraordinary challenge. This scenario is ideal to explore what Spain has done in terms of promotion and development of talent in women’s canoeing for their first Olympic participation. However, there is a clear lack of instruments that allow any type of trial in this discipline, since it is one of the most minority sports. Thus, this study aims to assess the psychometric properties and factorial structure of the International Canoeing Federation (ICF) questionnaire [28] to evaluate canoeists’ perceptions of the sport system’s capacity for talent development of women’s canoeists in Spain, to establish a free to use scale that allows generation of different strategies and lines of action for any of the professionals and organizations involved in this sport practice.

## 2. Materials and Methods

### 2.1. Instruments and Participants

A 12-question analytical survey was conducted. The first four questions capture the socio-demographic characteristics of the respondents, and the second block of eight items queries perceptions of talent development in women’s canoeing based on the ICF’s “Evaluation of the current position in canoeing-sport with regard to talent” questionnaire [28]. This questionnaire was developed by Szanto in 2010, a Hungarian author with expertise in canoeing. This tool is provided in the manuals of sport technicians given by the IFC for talent detection. Previously, it has not been evaluated by the scientific literature, so there is a need for its validation. Each score obtained is based on a Likert scale (1–5): 1 is “Strongly disagree”, 2 “Disagree”, 3 “Neither agree nor disagree”, 4 “Agree” and 5 “Strongly agree”. The survey was carried out with the collaboration of the Spanish Canoe Federation (RFEP), and 15 of the 17 Spanish Autonomous Communities participated, with a representation of 73 clubs. The total sample amounted to 167 participants. The inclusion criteria for this survey were women and men linked to still water canoeing (paddlers, coaches, technicians, referees, club managers, FFAA, RFEP, etc.), and of legal age (>18 years old) who had at some point practiced this sport.

Of the 167 participants, 62.87% were men and 37.13% women. The average age was 39 years, with the youngest participants being 18 and the oldest 68. Table 1 shows the characterization of the participants.

### 2.2. Procedure

An email was sent to the RFEP and distributed by the same, providing information on the purpose of the research, written informed consent and the URL to fill the questionnaires. The average time needed to answer the questionnaire was approximately 10 min. Data collection was performed using the Google Forms application, as electronic questionnaires have been proven to save costs and obtain higher participation [29]. The responses obtained were stored in a spreadsheet, facilitating their transformation and statistical analysis. Data collection was carried out between April and May 2020 (before the Olympic Games).

### 2.3. Data Analysis

Taking into account the ordinal character of the scale and of the information extracted from it employing a Likert scale, the free statistical package FACTOR (v.10.10.02, Rovira I Virgili University: Tarragona, Spain) [30] was used for the exploratory factor analysis (EFA) as the Promin method [31] for factor extraction. Robust unweighted least squares (RULS) [32] method for factor model definition and polychoric correlation matrix were used due to low normality values (*p* = 0.013) [33]. The suitable number of dimensions was determined through the best use of parallel analysis [34]. As sampling adequacy criteria, the Kaiser–Meyer–Olkin (KMO) and Bartlett’s tests of sphericity were selected [35].

The confirmatory factor analysis (CFA) was then carried out using the software package AMOS v.26.0.0 (IBM Corporation, Wexford, PA, USA). The components with weights under 0.60, cross loads greater than 0.40 and communalities less than 0.30 were discarded [36]. The following indices were used to assess the model’s goodness-of-fit: (1) the chi-squared probability setting (*p* > 0.05) [37]; (2) the comparative fit index (CFI) and (3) the non-normed fit index (NNFI) [38]; (4) the root mean square error of approximation (RMSEA) [38]; (5) the root mean square of residuals (RMSR) [39]; and (6) the chi-square per degree of freedom proportion (CMIN/DF) [40]. McDonald’s Omega was also chosen as the parameter for evaluating the questionnaire’s final structure [41], calculated using the software SPSS v.23.0.0 (IBM Corporation, Armonk, NY, USA).

## 3. Results

Table 2 shows the descriptive statistics of each item included in the original exploratory analysis.

The RULS method extracted a monofactorial structure for the questionnaire through the explained variance based on eigenvalues [42] (Table 3) and the reliability of expected a posteriori (EAP) scores [43].

Thus, no rotation method was selected due to the one-dimensional nature. The viability of the EFA was analyzed by the sampling adequacy indices that provided good results (KMO test = 0.771 and Bartlett test = 478.2; df = 28; *p* = 0.000). Table 4 reflects the loading matrix for eight items and one factor.

Following the EFA, item 8 was excluded since its load was less than 0.60 and its Kaiser’s single-variable measure of sampling adequacy (MSA) values were below 0.50. Consequently, a factor structure of seven items grouped in one dimension was established. Table 5 indicates the polychoric correlation matrix that defines the composition of the questionnaire.

**Figure 1:**
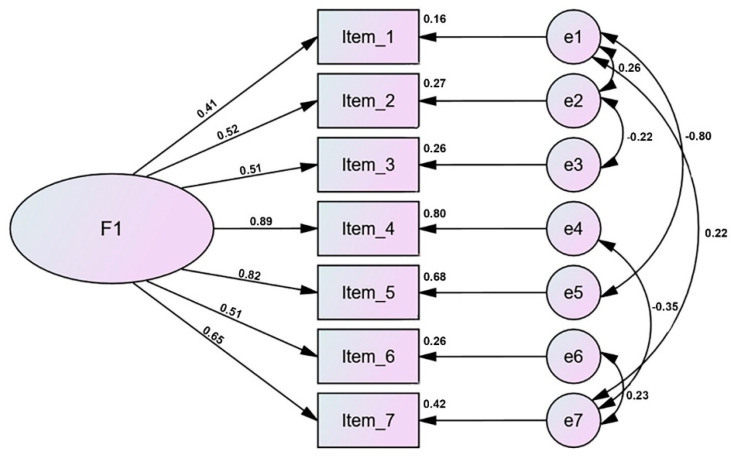
Factor model.

Once the EFA was carried out and the structure of the scale was defined, the CFA was conducted to assess the characteristics of the model (Figure 1).

Table 6 shows the goodness-of-fit indices after developing the CFA from the structure obtained in the EFA. The values reported an exceptional matching between the data and the model [44]. NNFI and CFI over 0.95 represent an impeccable fit to the model. In addition, the CMIN/DF index below 2 could be defined as suitable and chi-squared probability with non-significant values is excellent. The RMSEA is between the appropriate limits and RMSR could be considered extraordinary, as the values are less than 0.08.

Table 7 reflects reliability indicators for the single-dimensional structure composed by seven items, evaluated by McDonald’s Omega.

## 4. Discussion

The current study’s main contribution is an examination of the questionnaire’s psychometric properties to assess canoeists’ perceptions of the Spanish sport system’s capacity for talent development in women’s canoeing, as well as the construct validity and reliability indicators for the ICF questionnaire in a sample of Spanish canoeists. The research found a unidimensional factor structure with seven items and optimal goodness-of-fit indicators. Additionally, McDonald’s Omega coefficient revealed a high level of consistency.

For several decades, talent management is a crucial aspect of administrative debate and organizational practices [45]. Employee talent is one of an organization’s most powerful intangible assets [46], therefore, resource-based organizations demonstrated that specific assets inside an organization can provide dynamic skills that can be translated into long-term competitive advantages, productivity and outstanding performance [47]. People, actions and business activities’ inbound and outward logistics are all part of the sports industry, making it a one-of-a-kind enterprise [48]. Therefore, taking a strategic human resource management approach is critical. According to Lopez and his colleagues [49], businesses increasingly need an organizational structure that encourages the creation and use of new knowledge. What is more, gender quotas have not gained enough interest as a talent management approach in the Asia Pacific area, according to Tatli and its partners [50]. They believe it is necessary and legitimate to explore gender quotas as part of the region’s talent management plan to delve into untapped female potential to overcome talent shortages.

Regarding the results extracted from the scale, the great majority of the scores obtained in the sample report are close to 3 (neither agree or disagree), showing the need perceived by the professionals of this modality to find different formulas to promote women’s sports, as previous research has already pointed out [51,52]. When it comes to decision-making positions, women are a minority in most sports and in all countries. Twenty of the 52 sports federations do not include women, and 46 of them have less than 25% female presence [53]. In terms of coaching, previous studies detected an under-representation of women in sports coaching [54], determined by the poor self-efficacy perception by women athletes during their competitive stage that minimizes their willingness to become a coach later on [55], preventing the transmission of this knowledge to new generations of competitors. In sports development, and leaving aside the obvious and well-documented developmental characteristics that differentiate men and women [56], the gender data gap represents an unequal representation of women across multiple areas [57], hindering the generation and application of valid knowledge in the development of female athletes. Considering that public organizations play a fundamental role in the development of women’s sports at all levels and that the presence or absence of women in sports activities is a global indicator of their status in society [58], the incorporation of women in these positions is still very low in comparison with the opposite gender explaining the lack of actions and statutes that greatly promote the development of women’s sports [59].

Regarding the competition area, it should be understood that conception varies between genders, diminishing the sense of community in women [60], showing higher levels of competitive anxiety and coping differently with sport performance-related stress [61]; it is therefore implied that it is necessary to remodel both the structure and the competition system [62]. If the identification and development of talent in women’s sports is monitored, there are several studies which focused on talent opportunity programs only in those sports that are characterized by a high female participation [63,64]. Finally, there are many barriers for young girls who want to take up a particular sport, such as the inexistence of positive role models, insufficient opportunities and the bad quality of sports offered [65]. In addition, the environment must be considered, defined as social barriers to both the influences [66] and the evaluation of their close environment [67].

There are various limitations to this study. The number of participants is restricted, also the return rate of the emails sent is still unknown. The analysis presents a one-dimensional structure with six covariances relating the items, which means the existence of more than one latent variable, so the conclusions of this study should be taken with caution. This is preliminary work, as instrument validation is a process that takes time to develop; more evidence of the ICF’s psychometric qualities will be needed in the future. Therefore, future research should greatly increase the sample size, analyzing people related to this modality in different countries and including a larger number of items.

Moreover, this investigation does not use direct data collection techniques rather than telephone or internet questionnaires, which provide more accurate findings [29]. Online surveys, on the other hand, provide the benefits of lowering expenses, moving the interviewer in relation to the respondents, widening the sample, and simplifying data collecting and processing from the researcher’s perspective. Recruiting a larger sample size as a future line of research would be interesting to gain further data on the ICF’s merits. The ICF could be used at the whole context of the canoeing-sport, and it may be interesting for federations to analyze the beliefs of their professionals and develop new lines of action regarding sports talent.

## 5. Conclusions

People involved in the sport of canoeing identify serious needs in terms of detection and development of female talent at various scales, pointing out the importance of developing different lines of action at the institutional and sports level by all the people involved in the decision-making process.

The present study represents a contribution to the literature by providing the validation of a questionnaire that, in a very simple way, allows a perceived first look at key aspects in the production of talent within a sport system, such as the training of coaches, the quality of the initiation and talent recruitment systems, the suitability of competition programs and the synergies within the different levels of sport.

Therefore, this research provides a starting point for studies on the effectiveness and efficiency of sport systems in different contexts. Thus, future research should increase the knowledge in reference to this type of methodological tool that allows us to observe the intricacies of the sports system and characterize them, enabling the approach of improvements that help the modality to have a greater visibility and a greater overall development.

## Figures and Tables

**Table 1:** Characteristics of the sample (*n* = 167).

Variable	Categories	*n*	%
Sex	Male	105	62.9
	Female	62	37.1
Age	Under 30	101	60.5
	Between 30 and 40	25	15
	Between 41 and 50	31	18.6
	Over 50	10	5.9
Modality	Kayak	107	64.1
	Canoe	60	35.9
Active in sports	Yes	154	98.2
	No	13	7.8

**Table 2:** Descriptive statistics of items.

Item	Mean	SD	Variance
1	3.606	1.054	1.113
2	2.846	1.085	1.178
3	2.973	0.947	0.899
4	3.353	0.990	0.982
5	3.280	0.983	0.968
6	3.206	0.957	0.917
7	2.840	0.948	0.900
8	2.873	1.119	1.252

SD: Standard Deviation.

**Table 3:** Items explained variance based on eigenvalues.

Variable	Eigenvalue	Proportion of Variance
1	3.382	0.483
2	0.959	0.137
3	0.820	0.117
4	0.705	0.100
5	0.510	0.072
6	0.389	0.055
7	0.232	0.033
8	0.160	0.020

**Table 4:** Loading matrix.

Item	Factor
Do you consider that coaches have sufficient training to identify and develop athletes for women’s canoeing in Spain?	0.523
2. Do you consider the current systems for the initiation and development of women’s canoeing in your club to be effective?	0.591
3. Do you consider that there is a policy that links the Clubs–Autonomous Federations and the Royal Spanish Canoe Federation (RFEP) for the development of women’s canoeing in Spain?	0.520
4. Do you consider that the current competition programs are adequate to develop women’s canoeing?	0.872
5. Do you consider that the structure of the competition program encourages a progression in the development of canoe-women?	0.840
6. Do you consider the age at which female athletes are currently joining canoeing in Spain to be appropriate?	0.594
7. Do you believe that current talent recruitment strategies encourage the right type of athletes for women’s canoeing in Spain?	0.711
8. Do you consider that this sport has a low dropout rate of female canoeists in Spain?	0.081

Note: These items are a literal translation into English for ease of reading, not a cross-cultural adaptation into English.

**Table 5:** Polychoric correlation matrix for 7 items.

Items	1	2	3	4	5	6	7
1	1						
2	0.393	1					
3	0.258	0.113	1				
4	0.371	0.500	0.417	1			
5	0.288	0.392	0.440	0.738	1		
6	0.207	0.386	0.221	0.446	0.401	1	
7	0.399	0.282	0.398	0.460	0.537	0.480	1

**Table 6:** Goodness of fit indices.

Indices	Values
NNFI	0.971
CFI	0.992
CMIN/DF	1.322
Ρ (*χ*^2^)	0.227
RMSEA	0.046
RMSR	0.034

NNFI: non-normed fit index; CFI: comparative fit index; CMIN/DF: minimum discrepancy per degree of freedom; P (*χ*^2^): chi-squared probability; RMSEA: root mean square error of approximation; RMSR: root mean square of residuals.

**Table 7:** Reliability indices for each factor.

Indices	Values
McDonald’s Omega	0.816

## Data Availability

The datasets used during the current study are available from the corresponding author on reasonable request.

## References

[1] Thomson A., Schlenker K., Schulenkorf N. (2013). Conceptualizing Sport Event Legacy. Event Manag..

[2] Li S., Jago L. (2013). Evaluating Economic Impacts of Major Sports Events–a Meta Analysis of the Key Trends. Curr. Issues Tour..

[3] Cagigal J.M., Muros R.C., Hinkelbein F. (1979). Cultura Intelectual y Cultura Física.

[4] Szymanski S. (2003). Incentives and Competitive Balance in Team Sports. Eur. Sport Manag. Q..

[5] Sass M. (2016). Glory Hunters, Sugar Daddies, and Long-Term Competitive Balance under UEFA Financial Fair Play. J. Sports Econ..

[6] Gong H., Watanabe N.M., Brown M.T., Nagel M.S. (2019). The Impact of the Chinese Basketball Association’s Asian-Born Player Policy on Competitive Balance. J. Glob. Sport Manag..

[7] Scelles N. (2021). Policy, Political and Economic Determinants of the Evolution of Competitive Balance in the FIFA Women’s Football World Cups. Int. J. Sport Policy Politics.

[8] Bayle E., Madella A. (2002). Development of a Taxonomy of Performance for National Sport Organizations. Eur. J. Sport Sci..

[9] Winand M., Zintz T., Bayle E., Robinson L. (2010). Organizational Performance of Olympic Sport Governing Bodies: Dealing with Measurement and Priorities. Manag. Leis..

[10] O’Boyle I., Hassan D. (2014). Performance Management and Measurement in National-Level Non-Profit Sport Organisations. Eur. Sport Manag. Q..

[11] Torres L., Martin E., Guevara J.C. (2018). The Gold Rush: Analysis of the Performance of the Spanish Olympic Federations. Cogent Soc. Sci..

[12] De Bosscher V., De Knop P., Van Bottenburg M., Shibli S. (2006). A Conceptual Framework for Analysing Sports Policy Factors Leading to International Sporting Success. Eur. Sport Manag. Q..

[13] Ali M., Ullah M.S., Guha S. (2020). Role of Talent Development on Talent Engagement and Self-Efficacy: A Structural Model. J. Soc. Econ. Res..

[14] Ak E., Koçak S. (2010). Coincidence-Anticipation Timing and Reaction Time in Youth Tennis and Table Tennis Players. Percept. Mot. Ski..

[15] Dunwoody P.T. (2006). The Neglect of the Environment by Cognitive Psychology. J. Theor. Philos. Psychol..

[16] Muniesa C.A., González-Freire M., Santiago C., Lao J.I., Buxens A., Rubio J.C., Martín M.A., Arenas J., Gomez-Gallego F., Lucia A. (2010). World-Class Performance in Lightweight Rowing: Is It Genetically Influenced? A Comparison with Cyclists, Runners and Non-Athletes. Br. J. Sports Med..

[17] Chow J.Y., Davids K., Button C., Shuttleworth R., Renshaw I., Araujo D. (2006). Nonlinear Pedagogy: A Constraints-Led Framework for Understanding Emergence of Game Play and Movement Skills. Nonlinear Dyn. Psychol. Life Sci..

[18] Burillo P., Barajas Á., Gallardo L., García-Tascón M. (2011). The Influence of Economic Factors in Urban Sports Facility Planning: A Study on Spanish Regions. Eur. Plan. Stud..

[19] Escamilla-Fajardo P., Núñez-Pomar J.M., Prado-Gascó V. (2021). Economic Performance in Spanish Sports Clubs: Entrepreneurial Orientation of Professional and Non-Professional Teams Analysed through FsQCA. Eur. J. Int. Manag..

[20] España G. (1990). Ley 10/1990, de 15 de Octubre, Del Deporte. Madr. BOE.

[21] Gavala-González J., Castillo-Rodríguez A., Fernández-García J.C. (2019). Dual Career of the U-23 Spanish Canoeing Team. Front. Psychol..

[22] Anderson T., Kanuka H. (2002). E-Research: Methods, Strategies, and Issues.

[23] Collins M. (2014). Sport and Social Exclusion.

[24] Elling A., Knoppers A. (2005). Sport, Gender and Ethnicity: Practises of Symbolic Inclusion/Exclusion. J. Youth Adolesc..

[25] Pavlidis A., Connor J. (2016). Men in a ‘Women Only’ Sport? Contesting Gender Relations and Sex Integration in Roller Derby. Sport Soc..

[26] Acosta R.V., Carpenter L.J. (2014). Woman in Intercollegiate Sport: A Longitudinal, National Study. Thirty-Seven Year Update, 1977–2014. Acosta-Carpenter.

[27] Moawad J. (2019). Gender Inequality in Sports. FairPlay Rev. Filos. Ética Derecho Deporte.

[28] Szanto C. (2010). ICF Canoe Sprint Coaching Manual Level 2 and 3. Lausanne Int. Canoe Fed..

[29] Yeager D.S., Krosnick J.A., Chang L., Javitz H.S., Levendusky M.S., Simpser A., Wang R. (2011). Comparing the Accuracy of RDD Telephone Surveys and Internet Surveys Conducted with Probability and Non-Probability Samples. Public Opin. Q..

[30] Ferrando P.J., Lorenzo-Seva U. (2017). Program FACTOR at 10: Origins, Development and Future Directions. Psicothema.

[31] Lorenzo-Seva U. (1999). Promin: A Method for Oblique Factor Rotation. Multivar. Behav. Res..

[32] Paatero P. (1997). Least Squares Formulation of Robust Non-Negative Factor Analysis. Chemom. Intell. Lab. Syst..

[33] Holgado–Tello F.P., Chacón–Moscoso S., Barbero–García I., Vila–Abad E. (2010). Polychoric versus Pearson Correlations in Exploratory and Confirmatory Factor Analysis of Ordinal Variables. Qual. Quant..

[34] Hayton J.C., Allen D.G., Scarpello V. (2004). Factor Retention Decisions in Exploratory Factor Analysis: A Tutorial on Parallel Analysis. Organ. Res. Methods.

[35] Frías-Navarro D., Pascual-Soler M. (2012). Prácticas Del Análisi Factorial Exploratorio (AFE) En La Investigación Sobre Conducta Del Consumidor y Marketing. Suma Psicol..

[36] Hair J.F. (2010). Multivariate Data Analysis.

[37] Henseler J., Sarstedt M. (2013). Goodness-of-Fit Indices for Partial Least Squares Path Modeling. Comput. Stat..

[38] Xia Y., Yang Y. (2019). RMSEA, CFI, and TLI in Structural Equation Modeling with Ordered Categorical Data: The Story They Tell Depends on the Estimation Methods. Behav. Res..

[39] Kumar A., Sharma S. (1999). A Metric Measure for Direct Comparison of Competing Models in Covariance Structure Analysis. Struct. Equ. Modeling A Multidiscip. J..

[40] Wells C.S. (2021). Assessing Measurement Invariance for Applied Research.

[41] Kalkbrenner M.T. (2021). Alpha, Omega, and H Internal Consistency Reliability Estimates: Reviewing These Options and When to Use Them. Couns. Outcome Res. Eval..

[42] Chatelin F. (2012). Eigenvalues of Matrices: Revised Edition.

[43] Ferrando P.J., Lorenzo-Seva U. (2016). A Note on Improving EAP Trait Estimation in Oblique Factor-Analytic and Item Response Theory Models. Psicológica.

[44] Bentler P.M. (1990). Comparative Fit Indexes in Structural Models. Psychol. Bull..

[45] McDonnell A. (2011). Still Fighting the “War for Talent”? Bridging the Science Versus Practice Gap. J. Bus. Psychol..

[46] Wuen C.H., Ibrahim F., Ringim K.J. (2020). The Impact of Human Resource Management Practices on SMEs Performance: An Exploratory Study in Brunei Darussalam. Int. J. Asian Bus. Inf. Manag..

[47] Al Aina R., Atan T. (2020). The Impact of Implementing Talent Management Practices on Sustainable Organizational Performance. Sustainability.

[48] Bizen Y., Kishida K., Nogi S., Kawakami K., Yoshida H. (2018). Consciousness of Spending on Children’s Sports Activities in a Community Sports Club in Japan: Clarifying Parents’ Internal Reference Price. Int. J. Asian Bus. Inf. Manag..

[49] Vivas-López S., Peris-Ortiz M., Rueda-Armengot C. (2011). Managing Talent for Organisational Learning. Eur. J. Int. Manag..

[50] Tatli A., Vassilopoulou J., Özbilgin M. (2013). An Unrequited Affinity between Talent Shortages and Untapped Female Potential: The Relevance of Gender Quotas for Talent Management in High Growth Potential Economies of the Asia Pacific Region. Int. Bus. Rev..

[51] Folgar M.I., Lamas M.F., Fernández D.A., Salgado P.G., Boubeta A.R. (2019). Mujer y Piragua: Estudio de Las Variables Moduladoras Del Abandono Deportivo de Las Mujeres Piragüistas En Modalidades Olímpicas. Retos Nuevas Tend. Educ. Física Deporte Recreación.

[52] Talavera L.M., Saldaña L.M., Encinas V.G. Participation of Women in Adventure Sports. Proceedings of the 7th International Adventure Conference.

[53] European Commission (2014). Directorate General for Education and Culture. Gender Equality in Sport : Proposal for Strategic Actions 2014–2020.

[54] Walker N.A., Bopp T. (2011). The Underrepresentation of Women in the Male-Dominated Sport Workplace: Perspectives of Female Coaches. J. Workplace Rights.

[55] Moran-Miller K., Flores L.Y. (2011). Where Are the Women in Women’s Sports? Predictors of Female Athletes’ Interest in a Coaching Career. Res. Q. Exerc. Sport.

[56] Clarke A.C., Presland J., Rattray B., Pyne D.B. (2014). Critical Velocity as a Measure of Aerobic Fitness in Women’s Rugby Sevens. J. Sci. Med. Sport.

[57] Perez C.C. (2019). Invisible Women: Exposing Data Bias in a World Designed for Men.

[58] Monazami M., Alam S., Shetab Booshehri N. (2011). Determining the Factors Affecting the Development of Physical Education and Women’s Sports of the Islamic Republic of Iran. Sport Manag..

[59] Pfister G., Radtke S. (2009). Sport, Women, and Leadership: Results of a Project on Executives in German Sports Organizations. Eur. J. Sport Sci..

[60] Pretty G.M., McCarthy M. (1991). Exploring Psychological Sense of Community among Women and Men of the Corporation. J. Community Psychol..

[61] Harwood C., Cumming J., Fletcher D. (2004). Motivational Profiles and Psychological Skills Use within Elite Youth Sport. J. Appl. Sport Psychol..

[62] Warner S., Dixon M.A. (2015). Competition, Gender and the Sport Experience: An Exploration among College Athletes. Sport Educ. Soc..

[63] Sands W.A. (2013). Talent Identification and Development in Women’s Artistic Gymnastics: The Talent Opportunity Program (TOPs). Talent Identification and Development in Sport.

[64] Baker J., Cobley S., Schorer J. (2012). Talent Identification and Development in Sport: International Perspectives. Int. J. Sports Sci. Coach..

[65] Staurowsky E.J., De Souza M.J., Miller K.E., Sabo D., Shakib S., Theberge N., Veliz P., Weaver A., Williams N.I. (2015). Her Life Depends on It III: Sport, Physical Activity, and the Health and Well-Being of American Girls and Women. Women’s Sports Found..

[66] Coleman L., Cox L., Roker D. (2008). Girls and Young Women’s Participation in Physical Activity: Psychological and Social Influences. Health Educ. Res..

[67] Salvatore J., Marecek J. (2010). Gender in the Gym: Evaluation Concerns as Barriers to Women’s Weight Lifting. Sex Roles.

